# Oxidative Stress Response of Meagre to Dietary Black Soldier Fly Meal

**DOI:** 10.3390/ani12233232

**Published:** 2022-11-22

**Authors:** Inês Guerreiro, Carolina Castro, Cláudia R. Serra, Filipe Coutinho, Ana Couto, Helena Peres, Pedro Pousão-Ferreira, Laura Gasco, Francesco Gai, Aires Oliva-Teles, Paula Enes

**Affiliations:** 1 CIMAR/CIIMAR—Centro Interdisciplinar de Investigação Marinha e Ambiental, Universidade do Porto, Terminal de Cruzeiros do Porto de Leixões, Av. General Norton de Matos s/n, 4450-208 Matosinhos, Portugal; 2 Departamento de Biologia, Faculdade de Ciências, Universidade do Porto, Rua do Campo Alegre, Ed. FC4, 4169-007 Porto, Portugal; 3 Instituto Português do Mar e da Atmosfera (IPMA), Estação Piloto de Piscicultura de Olhão, Av. 5 de Outubro s/n, 8700-305 Olhão, Portugal; 4 Dipartimento di Scienze Agrarie, Forestali e Alimentari, Università degli Studi di Torino, Largo P. Braccini 2, 10095 Grugliasco, TO, Italy; 5 Istituto di Scienze delle Produzioni Alimentari, Consiglio Nazionale delle Ricerche, Largo P. Braccini 2, 10095 Grugliasco, TO, Italy

**Keywords:** *Argyrosomus regius*, glutathione, *Hermetia illucens*, insect meal, lipid peroxidation, oxidative stress enzymes

## Abstract

**Simple Summary:**

Given the need of replacing fishmeal with more sustainable ingredients, insect meal has appeared as a suitable alternative. As a result, in recent years, a large number of studies have emerged regarding the use of insect meal in diets for several fish species. However, chitin, a component of insect meal, is frequently pointed as a potential bottleneck for the inclusion of high levels of insect meal in aquafeeds. On the other hand, dietary chitin was also highlighted as having positive effects on fish antioxidant status. In fact, insect meal antioxidant potential can also be related with the insect’s nutritional composition, namely the lipid fraction. Nonetheless, few papers assessed the antioxidant effect of insect meal, particularly in marine fish species. The present study evaluated the effects of *Hermetia illucens* meal dietary inclusion on meagre liver and intestine oxidative status. The results show that with the tested *Hermetia illucens* meal levels, chitin or insect lipid composition does not greatly affect meagre liver or intestine oxidative status. Overall, it may be concluded that *Hermetia illucens* meal can be included as up to 30% of the diet without compromising meagre antioxidant status.

**Abstract:**

This study aimed to assess the effect of *Hermetia illucens* meal (HM) dietary inclusion on meagre oxidative status. Thus, fish were fed a fishmeal-based diet (CTR diet) and three other diets with increasing levels of HM inclusion, namely 10%, 20%, and 30% (diets HM10, HM20, and HM30, respectively). At the end of the trial, hepatic and intestine superoxide dismutase, catalase, and glucose-6-phosphate dehydrogenase activities and malondialdehyde concentration were unaffected by the diet composition. Liver glutathione peroxidase activity was higher in the fish fed the HM20 diet than in the fish fed the CTR and HM30 diets, and glutathione reductase activity linearly increased with the dietary HM level. The hepatic total glutathione and reduced glutathione contents were significantly lower in fish fed the HM20 diet than in fish fed the CTR and HM10 diets. In the intestine, the oxidized glutathione (GSSG) content and oxidative stress index linearly increased with the increase in dietary HM level, with the GSSG content of fish fed the HM20 diet being significantly higher than of fish fed the CTR diet. In conclusion, 30% HM might be included in meagre diets without negatively affecting hepatic and intestine oxidative status.

## 1. Introduction

In recent years, a large number of studies have emerged regarding the use of insect meal in diets for several fish species, including the meagre (*Argyrosomus regius*), a species with high potential for Mediterranean aquaculture diversification [1,2,3,4,5,6,7,8,9].

The maximum inclusion of insect meal in fish diets, without causing adverse effects on digestibility and growth performance, depends on the fish and insect species being evaluated [10,11]. Nonetheless, it is generally accepted that up to 30% of insect meal can be included in fish diets without causing adverse effects on growth and digestibility [3,4,10]. Insect meal has different chitin contents, which has frequently been pointed out as a potential bottleneck for the inclusion of insect meal in aquafeeds [5,12,13,14]. Notwithstanding, dietary chitin was also highlighted as having positive effects on fish antioxidant status [15,16].

In fact, insect meal antioxidant potential can be related not only to its chitin content but also to each insect’s nutritional composition, namely the lipid fraction, because lipids are the nutritional component most susceptible to oxidation [15]. Additionally, marine fish species can be more prone to oxidative stress than freshwater fish species, since the body lipid composition is different between both groups, with marine fish having a higher content of polyunsaturated fatty acids (PUFA) [17,18]. Although the effect of diets including insect meal on fish oxidative status has been assessed in several fish species [16,19,20,21,22], only two studies have been performed with marine species [23,24]. Shortly, in pearl gentian grouper (*Epinephelus lanceolatus* ♂ x *Epinephelus fuscoguttatus* ♀), up to 12.5% dietary defatted yellow mealworm (*Tenebrio molitor*) meal (TM) compromised liver antioxidant capacity, thus leading to a lower survival during a *Vibrio harvey* challenge [23]. In Japanese seabass (*Lateolabrax japonicus*), the inclusion of up to 19% defatted black soldier fly (*Hermetia illucens*) meal (HM) led to an improvement in fish serum antioxidant status [24]. As a consequence, there is a lack of studies on the antioxidant effect of insect meal, particularly in marine fish species.

The available literature points out that the effects of insect meal on fish oxidative status were mainly evaluated on the blood (serum or plasma) and liver [16,19]. However, the intestine, as the first tissue in contact with the feed, may be highly susceptible to oxidative stress when fish are fed diets including insect meal and, thus, deserves to be further studied [20,21,22,25,26].

*H. illucens* larvae are known to have a good nutritional profile [4,16]. Further, *H. illucens* larvae extracts were reported to present a complex of antioxidant compounds, thus making HM a feedstuff with antioxidant potential [27]. Accordingly, different studies showed an increase in antioxidant potential or a decrease in oxidative damage in fish fed with diets including HM compared to fishmeal-based diets [21,24,28,29]. For instance, African catfish (*Clarias gariepinus*) fed with 11.5% and 17% partially defatted HM showed an increase in serum catalase activity [29]. In Siberian sturgeon (*Acipenser baerii*), dietary inclusion of 37.5% highly defatted HM increased liver superoxide dismutase and glutathione reductase activities [28]. Further, tench (*Tinca tinca*) fed a diet including 11% full-fat HM and Japanese seabass fed diets including 5%, 10%, 14%, and 19% defatted HM had lower intestine and serum malondialdehyde, a marker of lipid peroxidation, respectively [21,24]. However, it was also shown in some species that dietary HM inclusion seemed to lead to oxidative status imbalances. For instance, rainbow trout (*Oncorhynchus mykiss*) fed 21% full-fat HM and Jian carp (*Cyprinus carpio* var. Jian) fed 8% and 11% defatted HM presented increased expression of stress biomarkers, namely heat-shock protein 70, thus suggesting a physiological activation of stress/inflammation response [30,31].

In meagre, 10% partially defatted HM inclusion had no negative effects on growth, digestibility, and overall health condition, namely distal intestine morphology, immune response, and gut microbiota composition. Nonetheless, higher inclusions, namely 20% and 30% HM, compromised growth and digestibility and led to an increase in overall intestine histomorphological alterations [5,6,9]. However, up to now, the effect of dietary HM inclusion on meagre oxidative status was not yet assessed. Thus, this study aimed to evaluate the effects of diets including up to 30% HM, replacing up to 52% of fishmeal, on meagre liver and intestine oxidative status.

## 2. Materials and Methods

### 2.1. Experimental Procedures

Four experimental diets were formulated to be isoproteic (50%) and isolipidic (19%). A fishmeal-based diet was used as a control (CTR diet), and 3 other diets were formulated to include 10%, 20%, and 30% of partially defatted black soldier fly (*Hermetia illucens*) larvae meal (HM) (diets HM10, HM20, and HM30, respectively), replacing 17%, 35%, and 52% of fishmeal, respectively. Dietary chitin content varied between 0.6% to 1.6%. The diets’ proximate composition, amino acid, and fatty acid composition are presented in Guerreiro et al. [5].

The experiment was conducted with meagre (*Argyrosomus regius*) juveniles with an initial mean body weight of 18.0 ± 0.02 g, and the growth trial is described in Guerreiro et al. [5]. Shortly, the experimental diets were assigned to triplicate groups of 18 fish, and the animals were fed by hand until visual satiation, twice a day, 6 days per week, for 9 weeks. At the end of the trial, 6 fish from each tank were randomly sampled 5 h after the morning meal, euthanized with a sharp blow to the head, and dissected on chilled trays for liver and whole-intestine sampling (3 fish for oxidative stress enzymatic activity assays and malondialdehyde concentration determination and 3 fish for glutathione measurement). Tissues were immediately frozen in liquid nitrogen and stored at −80 °C until analysis.

### 2.2. Enzymatic Activity Determination

Liver and intestine samples were homogenized (dilution 1:7 and 1:5, respectively) in ice-cold buffer (100 mM-Tris-HCl, 0.1 mM-EDTA, 0.1% Triton X-100 (*v*/*v*), pH 7.8). Homogenates were centrifuged at 30,000× *g* for 30 min at 4 °C, the resultant supernatant was collected, and aliquots were stored at −80 °C until analysis.

Superoxide dismutase (SOD; EC 1.15.1.1), catalase (CAT; EC 1.11.1.6), glucose-6-phosphate dehydrogenase (G6PD; EC 1.1.1.49), glutathione reductase (GR; EC 1.6.4.2), and glutathione peroxidase (GPX; EC 1.11.1.9) activities were measured as described by Castro et al. [32]. Activities are expressed as units (SOD and CAT) or milliunits (G6PD, GR, and GPX) per mg of soluble protein. One unit of SOD activity is defined as the amount of enzyme necessary to produce 50% inhibition of ferricytochrome C reduction rate. For the other enzymes, one unit of activity is defined as the amount of enzyme required to transform 1 μmol of substrate min^−1^ under the specific assay conditions. Protein concentration in the homogenates was determined according to Bradford [33] using Bio-Rad Protein Assay Dye Reagent (ref. 5 000 006, Amadora, Portugal) with bovine serum albumin as standard. All enzymatic assays were carried out at 37 °C in a Multiskan GO Microplate Reader (Model 5111 9200; Thermo Scientific, Nanjing, China).

### 2.3. Malondialdehyde (MDA) Determination

MDA concentration was used as a marker of lipid peroxidation. In the presence of thiobarbituric acid, MDA reacts, producing colored thiobarbituric acid reactive substances (TBARS), which were measured according to Buege and Aust [34]. Values are expressed as nmol MDA per g of wet tissue, calculated from a calibration curve.

### 2.4. Glutathione and Oxidative Stress Index

Liver and intestine samples were homogenized (dilution 1:5) in an ice-cold solution containing 1.3% 5-sulfosalicylic acid (w/v) and 10 mM HCl. Samples were centrifuged at 14,000× *g* for 10 min at 4 °C, and the supernatants were collected and stored at −80 °C until analysis.

Total glutathione (tGSH) and oxidized glutathione (GSSG) were quantified as described by Castro et al. [32]. Reduced glutathione (GSH) was calculated by the difference between tGSH and GSSG values.

The oxidative stress index (OSI) was calculated as follows:

OSI = 100 × (2 × GSSG/tGSH)

### 2.5. Statistical Analysis

All data were checked for normal distribution by the Shapiro–Wilk test and for homogeneity of variances by Levene’s test, normalized when appropriate, and analyzed by one-way ANOVA. A polynomial contrasts analysis was performed to determine whether the data followed a linear, quadratic, or cubic response to dietary HM inclusion. A significant level of 0.05 was used for the rejection of the null hypothesis. To illustrate the magnitude of the differences between means, a Tukey’s multiple range test was performed after ANOVA, when *p* < 0.05. All statistical analysis was done using SPSS 24.0 software package for Windows (IBM SPSS Statistics, New York, NY, USA).

## 3. Results

Meagre growth performance was not the aim of the present study, and such results are presented elsewhere [5]. Shortly, growth, feed, and protein efficiency ratios decreased with increased dietary HM inclusion, while feed intake was not affected by diet composition.

Regarding the present results, in the liver, SOD, CAT, and G6PD activities were unaffected by the experimental diets (Table 1). GPX activity was higher in fish fed with the HM20 diet than in fish fed the CTR and HM30 diets, and GR activity linearly increased with increased dietary HM level. The hepatic GSSG, OSI, and MDA contents were not affected by diet composition, while the tGSH and GSH contents were significantly lower in fish fed the HM20 diet than in fish fed the CTR and HM10 diets (Table 2).

At the intestinal level, no significant differences were observed in the antioxidant enzyme activities (Table 3). GSSG and OSI linearly increased with the increase in dietary HM level, while tGSH, GSH, and MDA were not affected by diet composition (Table 4). The GSSG content in fish fed the HM20 diet was significantly higher than in fish fed the CTR diet.

## 4. Discussion

When selecting an ingredient to use in animal feeds, besides assessing its potential effect on growth, it is also important that it will not compromise the animal’s health.

Insect meal was reported to have antioxidant properties related with its chitin or derivatives content [15]. Since black soldier fly also possesses this polysaccharide in its exoskeleton, the same properties are to be expected [5]. Accordingly, some studies showed that dietary inclusion of HM between 10–20% improved the serum and intestine oxidative status of Japanese seabass and tench, respectively [21,24], while other studies were unable to detect differences in oxidative status related to dietary inclusion of HM [28,29,35,36]. Still, other studies showed that similar dietary HM inclusion levels (10–20%) led to an activation of the stress/inflammation responses in Jian carp and rainbow trout [30,31].

In the present study, dietary HM inclusion up to 30% did not affect hepatic and intestine MDA levels, and only minor differences in GPX activity and glutathione content were observed.

The unsaturation degree of fatty acids directly impacts their susceptibility to oxidation, with unsaturated fatty acids being more prone to oxidation than the saturated (SFA) ones [17,37]. Insect fat is known to comprise a high level of SFA and monosaturated fatty acids (MUFA) [4], and, accordingly, the HM diets used in this study had a lower fatty acids unsaturation index than the fishmeal-based CTR diet [5]. This was also reflected in meagre whole-body fatty acid composition, which presented an unsaturation index that linearly decreased with the increase in dietary HM level [5]. The absence of a relation between whole-body fatty acid composition and lipid peroxidation susceptibility in meagre liver and intestine suggested that oxidative stress status was not affected by dietary HM lipid composition. Similarly, increased levels of SFA and decreased levels of PUFAs were also found in the body of Jian carp and muscle of rainbow trout fed HM, yet no effect on serum and liver MDA levels, respectively, was found [35,36].

The animals’ antioxidant defense system includes both antioxidant enzymes, namely SOD, CAT, GPX, and GR, and non-enzymatic antioxidants, such as glutathione and vitamins A and C. SOD is the first enzyme responding to the presence of oxygen radicals, catalyzing the dismutation of the superoxide anion O^2−^ to molecular oxygen and H_2_O_2_, thus preventing the radical chain reaction initiated by O^2−^. Thereafter, CAT or GPX reduce H_2_O_2_ to molecular oxygen and H_2_O [38].

Increased activity of SOD and CAT enzymes denotes an increase in the antioxidant potential, in an attempt to catalyze the oxygen radicals and avoid oxidative stress. In the present study, liver and intestine SOD and CAT activities were not affected by diet composition, which is in accordance with the observed identical MDA levels among diets.

As in this study, MDA content and SOD and CAT activities were not affected in the serum of Jian carp fed 3% and 5% defatted HM [30] nor in the liver of rainbow trout fed 20% and 40% partially defatted HM [35]. CAT activity and MDA content were also not affected in the liver of Siberian sturgeon fed 18.5% and 37.5% highly defatted HM [28]. As already discussed above, dietary HM lipid composition seemed to not affect meagre oxidative status.

Additionally, another aspect of the HM composition that might affect fish oxidative status is the HM chitin and derivatives content [15]. Thus, the unaffected SOD and CAT activities might be related with dietary chitin levels, which might be not high enough to affect the oxidative stress enzymes’ activities. As in the present study, where the chitin level of the diets varied between 0.6–1.6% [5], Elia et al. [35] reported chitin values of 1.05% and 2.09%, and Caimi et al. [28] reported values of 0.72% and 1.92%, suggesting that, until 2.09% of chitin in the diet, no major effects on CAT activity are observed. However, it is important to mention that Siberian sturgeon fed the higher HM level, 37.5%, corresponding to 2.09% chitin, presented increased liver SOD activity [28]. Additionally, increased CAT activity was observed in African catfish fed 35% full-fat *Gryllus bimaculatus*, which might be related to the higher level of chitin present in that diet, namely 2.7% [39]. Similarly, increased serum CAT activity was observed in African catfish fed 11.5% and 17% partially defatted HM [29] and in Jian carp fed 8% and 11% defatted HM [30]. However, none of the mentioned studies presented dietary chitin levels, so a direct comparison is not possible. As a major factor in the antioxidant potential of insect meal, future studies concerning these feedstuffs should report dietary chitin levels. Nonetheless, differences between works can also be attributed to insect meal nutritional composition and inclusion level, fish species, and size [40]. Since, depending on the incorporation level and the analyzed enzyme, the reported oxidative status might differ, it is important to evaluate not only the antioxidant potential but also the oxidative damage, to accurately identify the most appropriate insect meal quantity to add to aquafeeds.

The liver is the main producer and supplier of GSH for other tissues, which is related to its capability to convert methionine to cysteine, the rate-limiting precursor for GSH biosynthesis [41,42]. In accordance, in the present study, a higher GSH level was observed in the liver compared to the intestine. Similar observations were made in other fish species, for instance, gilthead seabream (*Sparus aurata*) and European sea bass (*Dicentrarchus labrax*) [32,43,44].

The effect of GPX on H_2_O_2_ detoxification is of fundamental importance in the protection of cells from oxidative damage [45]. GSH/GSSG ratio maintenance is achieved by the continuous reduction of GSSG to GSH, catalyzed by GR. Thereafter, GSH is required for GPX to reduce H_2_O_2_. Moreover, GSH might also directly scavenge ROS, and, thus, GSH deficiency contributes to oxidative stress [41]. In the present study, the increase in liver GPX activity in fish fed the HM20 diet led to a depletion in the GSH level, which was probably used by GPX in the process of H_2_O_2_ reduction. However, no relation could be established between the liver’s GSH and GSSG contents nor with the linear increase in the GR activity with the HM level, which did not lead to an increase in the GSH level. Likewise, no relation could be established between the observed linear increase in intestine GSSG content with dietary HM increase, and GSH content or GR activity, since no effect was observed on those parameters in the intestine. The higher liver GPX activity in fish fed the HM20 diet might indicate a higher level of H_2_O_2_ and, thus, a higher oxidative stress in those fish, although it was not enough to induce changes in the MDA levels. Moreover, CAT activity, the other enzyme responsible for H_2_O_2_ detoxification, was also not affected. The fact that, in the present study, an effect in the liver’s GPX activity and not in CAT activity was observed might be related with the level of H_2_O_2_ being produced and the consequent peroxidation route used. CAT is known to be more active when H_2_O_2_ production is higher, while GPX is stimulated when the level of H_2_O_2_ is lower [38]. This might suggest that the H_2_O_2_ produced in meagre fed the HM20 diet is higher than in the other diets, though not high enough to increase CAT activity.

Contrary to our results, Elia et al. [35] found lower liver and kidney SeGPX activity (the same GPX as the one analyzed in the present study) in rainbow trout fed 40% or 20% and 40% partially defatted HM, respectively, and hypothesized that it could be related to the different amino acid profiles of HM and fishmeal. The SeGPX enzyme’s GSH binding site contains one lysine and four arginine residues, and, thus, the lower level of those amino acids in HM diets, when compared to fishmeal, might have contributed to reduce SeGPX’s enzymatic efficiency [35]. In accordance, the HM diets used in the present study also presented lower levels of lysine and arginine, when compared to the fishmeal diet [5]. However, such a connection cannot be made, since, instead of decreasing, GPX activity increased in the liver of meagre fed the HM20 diet, and the activity in fish fed the other diets was not different from fish fed the CTR diet. Moreover, the decrease in GPX activity in the liver of Siberian sturgeon fed 37.5% highly defatted HM and rainbow trout fed 40% partially defatted HM was also related with dietary chitin level, which was 1.92% and 2.09%, respectively [28,35]. Chitin has the capability to bind selenocysteine, which is the catalytic center of SeGPX, making the selenocysteine unavailable for SeGPX activity [28,35,46,47]. Differences between those studies and the present results might be related to the higher HM dietary inclusion (37.5% and 40% vs. up to 30%), and consequent chitin levels (1.92% and 2.09% vs. 0.6–1.6%).

On the other hand, in the present study, GPX in the intestine was not affected by HM dietary inclusion. Similarly, GPX activity in tench intestine was not affected by 5.6% and 10.9% full-fat HM nor by 5.1% and 10.7% full-fat TM [21]. Similarly, 4% to 24.5% defatted TM did not affect GPX activity in largemouth bass (*Micropterus salmoides*) intestine [20].

The intestine as the main organ responsible for digestion, absorption, and metabolism might be more susceptible to dietary changes, being a key producer of oxygen radicals, thus playing an important role in the first stages of oxidative stress [48]. Despite the fact that no major effects on intestine antioxidant status were found, a linear increase in OSI with HM dietary increase might indicate some imbalance in the intestine’s redox status. In fact, the HM30 diet leads to an increase in meagre intestine histomorphological alterations [9]. However, no effect on the MDA level was detected.

## 5. Conclusions

In conclusion, although chitin has been reported to possess antioxidant potential, the present results showed that at the tested HM levels, chitin (until 1.6%) [5] does not greatly affect meagre liver or intestine oxidative status. On the other hand, it might be concluded that despite the observed decrease in growth performance, diet digestibility, and the increase in overall intestine histomorphological alterations in meagre fed 20% and 30% HM [5,6,9], those incorporation levels do not compromise meagre antioxidant status. Whole-body fatty acid profile changes due to feeding meagre with HM [5] also did not impact fish susceptibility for oxidative stress.

## Figures and Tables

**Table 1:** Hepatic levels of superoxide dismutase (SOD) and catalase (CAT) (U mg protein^−1^), glucose-6-phosphate dehydrogenase (G6PD), glutathione reductase (GR), and glutathione peroxidase (GPX) (mU mg protein^−1^) activities in meagre juveniles fed the experimental diets with different levels of *Hermetia* meal (HM).

	Diets				One-Way ANOVA	Polynomial Contrasts		
	CTR	HM10	HM20	HM30	*p*-Value	Linear	Quadratic	Cubic
SOD	91 ± 24	100 ± 34	98 ± 34	95 ± 32	0.930	0.831	0.562	0.813
CAT	320 ± 95	284 ± 47	303 ± 37	259 ± 42	0.189	0.079	0.848	0.218
G6PD	72 ± 6	77 ± 10	73 ± 16	85 ± 20	0.236	0.132	0.442	0.265
GR	5.2 ± 0.51	5.6 ± 0.77	5.6 ± 0.92	6.3 ± 1.27	0.080	0.016	0.616	0.435
GPX	174 ± 35 ^a^	193 ± 27 ^ab^	229 ± 23 ^b^	158 ± 49 ^a^	0.002	0.817	0.001	0.028

Mean values and standard deviation (±SD) are presented for each parameter (*n* = 9). Different letters in the same row stand for statistical differences between diets (*p* < 0.05).

**Table 2:** Hepatic levels of total glutathione (tGSH), reduced glutathione (GSH), and oxidized glutathione (GSSG) (mmol g tissue^−1^), oxidative stress index (OSI, %), and malondialdehyde (MDA, nmol malondialdehyde g tissue^−1^) in meagre juveniles fed the experimental diets with different levels of *Hermetia* meal (HM).

	Diets				One-Way ANOVA	Polynomial Contrasts		
	CTR	HM10	HM20	HM30	*p*-Value	Linear	Quadratic	Cubic
tGSH	1042 ± 91 ^b^	1041 ± 81 ^b^	905 ± 93 ^a^	972 ± 86 ^ab^	0.006	0.015	0.264	0.015
GSH	1038 ± 91 ^b^	1037 ± 81 ^b^	902 ± 92 ^a^	968 ± 86 ^ab^	0.006	0.015	0.263	0.016
GSSG	3.4 ± 1.48	3.7 ± 1.77	3.4 ± 1.32	3.7 ± 2.12	0.980	0.833	0.956	0.714
OSI	0.66 ± 0.30	0.71 ± 0.32	0.76 ± 0.27	0.77 ± 0.49	0.912	0.492	0.871	0.935
MDA	16 ± 5	22 ± 13	19 ± 11	28 ± 15	0.164	0.064	0.603	0.234

Mean values and standard deviation (±SD) are presented for each parameter (*n* = 9). Different letters in the same row stand for statistical differences between diets (*p* < 0.05).

**Table 3:** Intestine levels of superoxide dismutase (SOD) and catalase (CAT) (U mg protein^−1^), glucose-6-phosphate dehydrogenase (G6PD), glutathione reductase (GR), and glutathione peroxidase (GPX) (mU mg protein^−1^) activities in meagre juveniles fed the experimental diets with different levels of *Hermetia* meal (HM).

	Diets				One-Way ANOVA	Polynomial Contrasts		
	CTR	HM10	HM20	HM30	*p*-Value	Linear	Quadratic	Cubic
SOD	333 ± 101	312 ± 78	271 ± 55	279 ± 129	0.498	0.169	0.654	0.619
CAT	46.8 ± 28.2	71.7 ± 26.1	54.9 ± 17.6	52.3 ± 20.9	0.786	0.354	0.928	0.688
G6PD	3.28 ± 1.08	4.11 ± 1.09	4.39 ± 2.16	4.41 ± 1.59	0.227	0.150	0.209	0.394
GR	7.38 ± 1.92	7.38 ± 1.29	7.38 ± 0.39	8.55 ± 1.03	0.838	0.390	0.883	0.801
GPX	47 ± 13.4	36.7 ± 3.9	39.2 ± 13.6	46.8 ± 12.5	0.311	0.985	0.063	0.969

Mean values and standard deviation (±SD) are presented for each parameter (*n* = 9).

**Table 4:** Intestine levels of total glutathione (tGSH), reduced glutathione (GSH), and oxidized glutathione (GSSG) (mmol g tissue^−1^), oxidative stress index (OSI, %), and malondialdehyde (MDA, nmol malondialdehyde g tissue^−1^) in meagre juveniles fed the experimental diets with different levels of *Hermetia* meal (HM).

	Diets				One-Way ANOVA	Polynomial Contrasts		
	CTR	HM10	HM20	HM30	*p*-Value	Linear	Quadratic	Cubic
tGSH	845 ± 189	743 ± 158	792 ± 199	805 ± 232	0.745	0.815	0.386	0.527
GSH	830 ± 189	725 ± 158	769 ± 197	780 ± 223	0.717	0.714	0.373	0.529
GSSG	14.7 ± 3.17 ^a^	16.6 ± 2.41 ^ab^	20.5 ± 5.45 ^b^	18.0 ± 2.61 ^ab^	0.021	0.023	0.106	0.150
OSI	3.67 ± 1.11	4.44 ± 0.54	5.37 ± 1.66	5.1 ± 1.49	0.045	0.014	0.255	0.500
MDA	102 ± 31	126 ± 48	103 ± 46	98 ± 35	0.475	0.601	0.285	0.302

Mean values and standard deviation (±SD) are presented for each parameter (*n* = 9). Different letters in the same row stand for statistical differences between diets (*p* < 0.05).

## Data Availability

The data used to generate the results in this manuscript can be made available if requested from the corresponding author.
